# The Inconsistency Between Women's Preference and Actual Mode of Delivery in China: Findings From a Prospective Cohort Study

**DOI:** 10.3389/fpubh.2022.782784

**Published:** 2022-03-30

**Authors:** Jing Wu, Li Feng, Hongwei Zhang, Li Guo, Rafael Pérez-Escamilla, Yifei Hu

**Affiliations:** ^1^School of Agroforestry & Medicine, the Open University of China, Beijing, China; ^2^Obstetrics Department, Beijing Obstetrics and Gynecology Hospital, Capital Medical University, Beijing, China; ^3^Yale School of Public Health, Yale University, New Haven, CT, United States; ^4^Department of Child, Adolescent health and Maternal health, School of Public Health, Capital Medical University, Beijing, China

**Keywords:** cesarean section, vaginal delivery, reproductive health, delivery preference, maternal obesity, amniotic fluid, inconsistency

## Abstract

**Background:**

Previous studies have found that the rates of cesarean preference were much lower than the actual rates of cesarean births in China. We aimed to observe this inconsistency between preferred and actual modes of delivery and the factors associated with the inconsistency.

**Methods:**

We conducted a prospective cohort study at the maternity hospital with the largest number of deliveries in Beijing. We collected data through a questionnaire applied in the outpatient department, and medical records from the hospital's information system. Unconditional logistic regression was used to identify factors influencing the inconsistency between preferred and actual delivery mode.

**Results:**

The rates of actual cesarean section and of cesarean preference were 41 and 17%, respectively (χ^2^ = 82.9, *P* < 0.0001). The overall inconsistency rate was 31%, with 119 women preferred vaginal delivery but experienced cesarean section, accounting for 67% women undergoing cesarean section. Risk factors for this inconsistency between preferred vaginal delivery and actual cesarean section included: maternal obesity, receiving assisted reproduction, having an abnormal amniotic fluid volume, and fetal distress. Pre-labor rupture of membranes was a unique factor associated with such inconsistency between cesarean section preference and vaginal delivery at delivery.

**Conclusions:**

The inconsistent rate between preferred delivery at late pregnancy and actual delivery is high in China. Further research is needed to understand how to lower cesarean rates in China, taking maternal preferences for vaginal deliveries into account.

## Introduction

Cesarean section (CS) rates have increased dramatically in recent years globally, with wide ranges across countries (1, 2), and with China having one of the highest CS rates in the world (3). Previous studies conducted in China have shown that the overall CS rate nationwide increased from 2% in the 1960–70s to 36.7% in 2018 (3, 4). Interestingly, there is wide geographical variation in CS rates within China (5). Indeed, the CS has kept in a downward tendency in metropolitan areas, while being accompanied increases and high CS rates in small and middle cities and rural areas (5, 6). As a result, the overall CS rate in China of 36.7% (4) is almost 4-fold the CS rate of 10% recommended by World Health Organization (WHO) (7), based on minimizing maternal and perinatal mortalities (8, 9).

The risk factors for CS vary across countries and across time within a country (10–13). These risk factors can be classified into four categories: maternal, maternal-fetal, fetal, and social. It is important to note that CS delivery on maternal request (CDMR) explains about 24% of all CS in China (14). Interestingly, primiparous women are now more likely to prefer a vaginal delivery (VD) than a CS delivery under the two-child policy (15). Furthermore, since 2011, the government of China has invested in maternal care monitoring systems to bring down the excessive CS rates (15, 16). This is likely to explain the CS declines in big cities of China. However, starting in 2016, which is the initial year of universal two-child policy, a non-significant increase was found in Wuhan, China (17).

As indicated above, maternal and fetal factors play important roles in the increases in CS rates globally. These risk factors include older maternal age, higher household income, having medical insurance, higher educational attainment, multiple pregnancies, large neonatal weight, maternal obesity, placenta previa, fetal malpresentation, and preterm delivery (3, 18). On the one hand, the preferred mode of delivery predicted the actual delivery route (19, 20). As expected, there was a higher cesarean rate in women preferring cesarean than in those preferring VD (21, 22). On the other hand, the CS preference rates were much lower than the actual CS rates (13, 21, 23). A study reported an increased preference for CS among Chinese women asked about it during pregnancy compare to after childbirth (10% vs. 28%) (24). This finding strongly suggests that the actual delivery of mode drove this preference change. Hence, the main objective of this prospective study is to identify the factors associated with the inconsistency between how the mothers initially prefer for their babies to be delivered and how they are actually given birth. We expect that findings from this study will inform policy to lower CS rate.

## Methods

This prospective study was conducted from April to September, 2014 in the largest maternity hospital in Beijing, which is a teaching hospital affiliated to Capital Medical University, and a WHO Collaborating Center for Research and Training. This study was reviewed and approved by the Institutional Review Board at Beijing Obstetrics and Gynecology Hospital, Capital Medical University, Beijing, China.

The participants were recruited in the outpatient departments of the hospital. The eligibility criteria for this study were: singleton pregnancy, maternal age ≥18 years, gestation age between 28 and 37 weeks, and delivery in the study hospital. We collected data using a self-administrated structured-questionnaire and by extracting medical records information from the hospital information system. Trained health staff interviewed the women and performed the clinical data extraction. Once the identified personal information was used to link the questionnaire with medical information data and merged into one file, it was de-identified for further data analysis.

The variables examined included socio-demographics (parity, gravidity, age, education, place of residence, household income, and medical insurance), and childbirth preference (preferring CS/preferring VD) (25). The clinical data included the actual mode of delivery, the indications for CS (including CDMR), the maternal height and weight before/on the day of delivery, gestational age at delivery, fetal presentation, neonatal weight, length of hospital stay, amniotic fluid volume, as well as the presence of postpartum hemorrhage, assisted reproduction, fetal dysplasia, placenta previa, pre-labor membrane rupture (PROM), hysteromyoma at pregnancy, fetal distress, gestational diabetes, gestational hypertension, and previous CS. Although CDMR was not encouraged in our study period, doctors respected the choice by women who requested CS and relaxed the indications of CS for them. Body mass index (BMI) upon enrollment was used to classify the pregnant women into four groups: underweight (< 18.5 kg/m^2^), healthy weight (18.5–23.9 kg/m^2^), overweight (24–27.9 kg/m^2^), obesity (≥ 28 kg/m^2^) (26). Preterm delivery was classified as a gestational age <37 weeks. Postpartum hemorrhage was defined as blood loss >500 ml within the first 24 h after delivery. Amniotic fluid volume was monitored and categorized as polyhydramnios, exceeding 2,000 ml; oligohydramnios, <300 ml; or normal volume, between 300 and 2,000 ml.

The inconsistency between the preferred and the actual delivery mode was defined as the change from preferring VD during the third trimester to actual CS at the delivery or vice versa. In contrast, the consistency was defined as no change between preferred and actual childbirth.

Statistical power was determined to be more than 95% based on a sample size of 400 women to detect a 5% difference of our primary outcome, based on the difference between preferred and actual delivery model (39.7%) (22). The data were analyzed using SAS 9.3 (SAS Institute Inc., Cary, North Carolina, USA). The McNemar test was used to compare CS rates between the two preference groups (i.e., preferring vaginal vs. CS delivery). We compared maternal and obstetric characteristics, maternal and neonatal outcomes between the consistent and inconsistent groups, stratified by delivery modes preference. We performed univariate logistic regression analyses to find associations between maternal and neonatal characteristics and the odds ratios (OR) of the inconsistency between the preferred and the actual delivery modes. Those variables significantly associated (P≤0.05) in univariate analyses were included in multivariate logistic regression with stepwise selection. Both *p*-values of entry and exit into the final model were set at 0.05.

## Results

In total, we approached 472 pregnant women in the outpatient obstetrics department, and enrolled 450 women into the study. After we excluded 18 women because of incomplete data, 432 women provided data for the analyses. Among them, 177 (177/432, 41.0%) delivered via CS, in spite that only 72 (72/432, 16.7%) preferred this delivery mode. Of the 177 women who had a CS, 50 (28.2%) experienced an emergency CS, and 56 (31.6%) had CDMR. There was no significant difference of CDMR rate between women preferring CS and VD (36.2 vs. 29.4%; χ^2^ = 0.83; *P* = 0.36). Those women with prior CS continued to have repeated CS. Among the 255 women who experienced a VD, 128 (50.2%) women had an episiotomy. The McNemar test shows that the rate of actual CS was significantly higher than the rate of preferred CS (χ^2^ = 82.89; *P* < 0.0001) (Table 1). The inconsistency rate between the preference of delivery mode and the actual delivery mode was 30.8% (133/432) among all women.

**Table 1:** Paired fourfold table of mode of delivery and preference.

**Mode of delivery**	**Preference for mode of delivery (n(%))**		**Total**
	**VD**	**CS**	
VD	241(66.9)	14(19.4)	255(59.0)
CS	119(33.1)	58(80.6)	177(41.0)
Total	360(83.3)	72(16.7)	432(100)

*CS, cesarean section; VD, vaginal delivery*.

Table 2 shows the different maternal and obstetric characteristics, and obstetric and neonatal outcomes according to the inconsistency between the actual mode of delivery and the initial maternal preference. Among women who preferred a VD, those ending up delivering via CS were more likely to: be older than 35 years, have obesity, have received assisted reproductive technology (ART) services, and to have abnormal amniotic fluid, breech presentation, and postpartum hemorrhage than those women in the consistent vaginal delivery group. Furthermore, women who preferred VD but ended up delivering via CS were less likely to experience PROM and have fetal distress than those who preferred and actually had a VD. There was a higher proportion of primiparous women among women who preferred CS but delivered vaginally. The other factors associated were similar between the inconsistent group and the consistent group, regardless of delivery mode preference.

**Table 2:** Maternal characteristics, obstetric characteristics, obstetric and neonatal outcomes stratified by preference for delivery mode and the inconsistency.

**Indication**	**Preference for VD (*****n*** **= 360) (n(%))**			**Preference for CS (*****n*** **= 72) (n(%))**		
	**The inconsistent group (CS) (*n* = 119)**	**The consistent group (VD) (*n* = 241)**	***P*-value**	**The inconsistent group (VD) (*n* = 14)**	**The consistent group (CS) (*n* = 58)**	***P*-value**
**Maternal characteristics**						
Primipara	102 (85.7)	217 (90.0)	0.22	14 (100.0)	41 (70.7)	0.05^a^^b^
Gravidity ≥2 times	56 (47.1)	89 (36.9)	0.07	7 (50.0)	45 (77.6)	0.08^b^
Maternal age ≥35 years	23 (19.3)	23 (9.5)	0.01^a^	2 (14.3)	22 (37.9)	0.17^b^
Outside Beijing of China	15 (12.6)	17 (7.1)	0.08	0	7 (12.1)	0.39^b^
>12 years of education	9 (7.6)	19 (7.9)	0.91	2 (14.3)	9 (15.5)	1^b^
Family income <5,000 RMB	15 (12.6)	31 (12.9)	0.94	1 (7.1)	8 (13.8)	0.82^b^
No medical insurance	20 (16.8)	31 (12.9)	0.31	2 (14.3)	13 (22.4)	0.76^b^
BMI≥28	65 (54.6)	82 (34.0)	0^a^	6 (42.9)	24 (41.4)	0.92
**Obstetric characteristics**						
ART	8 (6.7)	5 (2.1)	0.05^a^^b^	0	4 (6.9)	0.58^c^
Pregnancy with hysteromyoma	8 (6.7)	12 (5.0)	0.50	1 (7.1)	1 (1.7)	0.35^c^
Preterm at delivery (<37 weeks)	2 (1.7)	6 (2.5)	0.91^b^	1 (7.1)	4 (6.9)	1^c^
Gestational diabetes	30 (25.2)	67 (27.8)	0.60	6 (42.9)	16 (27.6)	0.43^b^
Gestational hypertension	8 (6.7)	21 (8.7)	0.51	1 (7.1)	8 (13.8)	0.82^b^
Polyhydramnios/oligohydramnios	9 (7.6)	1 (0.4)	0^a^^b^	1 (7.1)	6 (10.3)	1^b^
PROM	23 (19.3)	77 (32.0)	0.01^a^	7 (50.0)	7 (12.1)	0^a^^b^
Placenta previa	3 (2.5)	3 (1.2)	0.40^c^	0	3 (5.2)	0.90^c^
Fetal distress	23 (19.3)	88 (36.5)	0^a^	3 (21.4)	3 (5.2)	0.15^b^
Breech presentation	18 (15.1)	0	0^a^	0	5 (8.6)	0.58^c^
**Obstetric and neonatal outcomes**						
Fetal malformation	4 (3.4)	6 (2.5)	0.89^b^	0	5 (8.6)	0.58^c^
Low neonatal weight (<2,500 g)	1 (0.8)	4 (1.7)	1^c^	1 (7.1)	6 (10.3)	1^b^
Postpartum hemorrhage	46 (38.7)	24 (10.0)	0^a^	1 (7.1)	11 (19.0)	0.51^b^
Hospital stay≥4 days	75 (63.0)	82 (34.0)	0^a^	2 (14.3)	36 (62.1)	0^a^
Operative VD	-	123 (51.0)	-	5 (35.7)	-	-
CDMR	35 (29.4)	-	-	-	21 (36.2)	-
Emergency CS	42 (35.3)	-	-	-	8 (13.8)	-

*CS, cesarean section; VD, vaginal delivery; BMI, body mass index; ART, assisted reproductive technology; PROM, pre-labor rupture of membranes; CDMR, cesarean delivery on maternal request*.

a *P ≤ 0.05*.

b *Continuous adjustment χ^2^-test*;

c *Fisher exact test; others Pearson χ^2^-test*.

Table 3 presents the predictors of the inconsistency between preferred and actual delivery mode by three multivariable logistic models. Model 1 identified the predictors of the overall inconsistency between preferred and actual delivery mode among all women (*n* = 432): maternal obesity [adjusted odds ratio (aOR) = 2.10; 95% confidence internal (CI) = 1.38–3.21], abnormal amniotic fluid volume [aOR = 3.44; 95% CI = 1.26–9.56] and lack of fetal distress [aOR = 0.52; 95% CI = 0.31–0.86]. Model 2 identified the predictors of the change from preferred VD to actual CS among the 360 women who had a preference for VD: maternal obesity [aOR = 2.42; 95% CI = 1.50–3.90], had received ART [aOR = 4.01 (1.15–14.01)], abnormal amniotic fluid volume [aOR = 30.99; 95% CI = 3.65–263.50] and lack of fetal distress [aOR = 0.31; 95% CI = 0.17–0.56]. Model 3 identified that those women who had PROM were more likely to drive the inconsistency that happened from desiring a CS during late pregnancy to actually having a VD during childbirth, among the 72 women who expressed this preference for CS delivery [aOR = 7.28; 95% CI = 1.96–27.06].

**Table 3:** Predictors of the inconsistency between the preferred and the actual delivery mode among all women, or those who preferred for VD, or those who preferred for CS.

		**Preferring delivery mode**	
Predictors	1.All women^a^ (*n* = 432) aOR (95% CI)	2.preference for VD^b^ (*n* = 360) aOR (95%CI)	3. preference for CS^c^ (*n* = 72) aOR (95%CI)
BMI ≥28	2.10 (1.38–3.21)	2.42 (1.50–3.90)	-
ART	-	4.01 (1.15–14.01)	-
Polyhydramnios/oligohydramnios	3.44 (1.26–9.56)	30.99 (3.65–263.50)	-
PROM	-	-	7.28 (1.96–27.06)
Fetal distress	0.52 (0.31–0.86)	0.31 (0.17–0.56)	-

*CS, cesarean section; VD, vaginal delivery; aOR, adjusted odd ratios; BMI, body mass index; ART, assisted reproductive technology; PROM, pre-labor rupture of membranes*.

a *Gravidity, maternal age, outside of Beijing, BMI, ART, amniotic fluid volume, fetal distress and PROM were included in multivariable analyses*.

b *Gravidity, maternal age, outside of Beijing, BMI, ART, amniotic fluid volume, fetal distress and PROM were included in multivariable analyses*.

C *PROM was included in multivariable analyses*.

## Discussion

We found a big gap between the preference for CS and the actual prevalence of CS in China, which is consistent with other studies (21, 22, 24, 27). The inconsistency rate (31%) between preferred and actual delivery modes in this study was in between the rate of 21% in Henan (23) and 47% in Beijing (24). The number of women who preferred a VD but had CS was much larger than the number of women who wished to have a CS but ended up delivering vaginally. The predominance of the inconsistency being in the direction from VD preference to ending up having a CS may explain why CS rates continue to be high in China (e.g., far above 10%). Our study is unique in that it is based on a robust prospective study design to analyze risk factors for the delivery mode inconsistency. Furthermore, in this study we were able to carefully categorize women according to their preferred and actual delivery mode and if they were inconsistent or not.

The CS rate (41%) in our study was much higher than recommended by both WHO and the Chinese government. However, it was not as high as previously reported in other hospital-based studies in China where CS rates have been documented to be nearly 60% (28, 29). This may be in part because it was carried out in a WHO collaborating center hospital that admits more complicated cases and perhaps follows a stricter standard of CS indication than the hospitals included in other studies. Interestingly, in our study there was no significant difference in the CDMR rate among women who underwent CS regardless of their initial delivery mode preference.

The change from preferring a VD during the third trimester of pregnancy but ending up delivering via a CS may be explained by medical, social, cultural and individual factors (23, 27, 30, 31), and can also be influenced by family members and health professionals (32). In our study, we found several factors that were associated with the inconsistency from maternal characteristics, obstetric indications for delivery type, and neonatal outcomes.

The prevalence of maternal obesity in this study was high (41%), and the rate was higher in the inconsistent group than that in the consistent group among women who preferred a VD. Maternal obesity was also associated with the change from preferring a VD to ending up having a CS. A robust meta-analysis (33) found that a higher occurrence of large for gestational age or macrosomia, which may be a consequence of maternal obesity, might lead to a CS and negatively affect maternal and neonatal health. It is important to improve the management and control of overweight or obesity among women of child-bearing age. Maintaining normal BMI and weight gain during pregnancy have indeed been identified as a key measure to reduce CS among women who prefer a VD (33). Both maternal obesity and CS were higher than the threshold recommended by WHO for preventing adverse outcomes for mothers and their offspring in short or long term (34, 35). Therefore, obesity prevention or management prior to or during pregnancy needs to be addressed to keep mothers and children healthy and safe (33).

In our study, an abnormal amniotic fluid volume was also found to be a risk factor for the change from preferring a VD in pregnancy to undergoing a CS, which is consistent with previous studies (36–39). A study found that oligohydramnios pregnancies were more likely to end up with a CS compared with normal amniotic fluid pregnancies (47 *vs*. 17%, *P* < 0.001) (39). Doctors should inform women who have preference for VD to be prepared for possible CS if they have a polyhydramnios or oligohydramnios pregnancy.

In the above study (39), fetal distress was one of the main indications for CS, which is at odds with our findings. More women who didn't change from preferred VD to actual CS in our study. It might be ascribed to more fetal monitoring during vaginal birth which incurred more fetal distress reports in this study. Being a doctor in China, especially an obstetrician, is a high-risk occupation given the potential for tense relationships between doctors and patients that sometimes even lead to life threats from patients (40). Doctors try their best to avoid any risk for patients, especially those related to maternal and neonatal death, given the increased frequency of violence from patients against providers in clinical settings (41).

PROM was independently associated with the switch from preferring a CS to actually having a VD. However, PROM at term has been previously found to be a risk factor for CS (42), which is inconsistent with our study. This inconsistency may be due to different characteristics of the participants between the studies, including the proportion of nulliparous women expecting CS (42). It is worth to further study if and how PROM may impact CS in China across different population subgroups of women. This is because PROM can lead to intraamniotic infection, further cause neonatal mortality or morbidity (43). The mode of delivery for women with PROM should be decided based upon the safety for both, the infants and their mothers (44).

There were some limitations in this study. First, a quantitative study is hard to make further interpretation of the inconsistency. In future, we may adopt a mixed methodology, for instance, conduct a qualitative interview among those mind-changers. Second, we conducted the study in a maternity hospital in Beijing; therefore, the findings cannot be generalized to throughout China, but which could be index indicators of pregnant women's preference and its impact on delivery mode. Since the study was conducted several years before, the current situation related to preferred vs. actual delivery mode may have changed, especially in the context of the COVID-19 pandemic. However, the newly released census report in the context of third-child policy implemented in 2021, indicates that our study is still valuable for informing delivery policies in China.

## Conclusions

The rate of CS was excessive in this study, and one-third of the women did not deliver their babies according to their delivery mode preferences. The inconsistency analyses conducted in our study can help screen for women who prefer a VD but that are at risk of ending up having a CS delivery even though there may not be a valid medical indication for it. Hence our findings can inform the design of interventions for lowering CS rates in China in the context of the “three-child” policy.

## Data Availability Statement

The raw data supporting the conclusions of this article will be made available by the authors, without undue reservation.

## Ethics Statement

The studies involving human participants were reviewed and approved by the Institutional Review Board at Beijing Obstetrics and Gynecology Hospital, Capital Medical University, Beijing, China. Written informed consent for participation was not required for this study in accordance with the national legislation and the institutional requirements.

## Author Contributions

JW, HZ, and YH designed the study. HZ and LG collected the data. JW and LF analyzed the data. JW, YH, LF, and RP-E wrote and revised the manuscript. All authors approved the final manuscript.

## Funding

This work was supported by Beijing Obstetrics and Gynecology Hospital, Capital Medical University [fcyy201413]. YH was supported in part by National Natural Science Foundation of China [82073574 and 81673232].

## Conflict of Interest

The authors declare that the research was conducted in the absence of any commercial or financial relationships that could be construed as a potential conflict of interest.

## Publisher's Note

All claims expressed in this article are solely those of the authors and do not necessarily represent those of their affiliated organizations, or those of the publisher, the editors and the reviewers. Any product that may be evaluated in this article, or claim that may be made by its manufacturer, is not guaranteed or endorsed by the publisher.
